# Incidence and prevalence of clinically relevant pituitary adenomas: retrospective cohort study in a Health Management Organization in Buenos Aires, Argentina

**DOI:** 10.1590/2359-3997000000195

**Published:** 2016-08-23

**Authors:** Patricia Fainstein Day, Monica Graciela Loto, Mariela Glerean, María Fabiana Russo Picasso, Soledad Lovazzano, Diego Hernán Giunta

**Affiliations:** 1 Department of Endocrinology and Nuclear Medicine Hospital Italiano de Buenos Aires Buenos Aires Argentina Department of Endocrinology and Nuclear Medicine, Hospital Italiano de Buenos Aires, Buenos Aires, Argentina; 2 Research in Internal Medicine Unit Department of Internal Medicine Hospital Italiano de Buenos Aires Buenos Aires Argentina Research in Internal Medicine Unit, Department of Internal Medicine, Hospital Italiano de Buenos Aires, Buenos Aires, Argentina

**Keywords:** Incidence and prevalence, pituitary adenomas

## Abstract

**Objectives:**

The main purpose of this study was to estimate the incidence rate and prevalence of clinically relevant pituitary adenomas (PAs) within the Hospital Italiano Medical Care Program (HIMCP), a well-defined population of 150,000 members living in the urban and suburban area of the city of Buenos Aires. We defined clinically relevant PAs as those associated with endocrine dysfunction and/or mass effect.

**Subjects and methods:**

A retrospective open cohort study was conducted, including all members of the HIMCP over 18 years old, with active memberships during the period of the study, from January 1^st^ 2003, to January 1, 2014. The incidence rates (IRs) were standardized (SIR) to the World Health Organization (WHO) 2000 standard population and were expressed per 100,000 members/year. Prevalence was estimated at January 1, 2014, and was expressed per 100,000 persons. The clinical records have been electronically managed since 2001. All lab and imaging studies were done in-house.

**Results:**

The overall SIR was 7.39/100,000/year (95% CI 4.47-10.31). Female patients had a specific IR significantly higher than male patients (5.85 vs.1.54) and represented 73% of the affected members. Regarding tumor size, 61.4% were microadenomas, and the mean age at diagnosis was 46.4 years. Prolactinomas had the highest SIR (5.41), followed by acromegaly (Acro) and non-functioning adenomas (NFAs) with overlapping 95% CIs (0.44-1.41 and 0.31-0.99, respectively). Microprolactinomas were more frequent in female (72.6%) (p < 0.01) and younger members (38 vs.60 years; p < 0.04). The overall prevalence rate was 97.76/100,000. Prolactinomas had the highest prevalence (56.29), followed by NFAs (21.48), Acro (14.07) and CD (5.93).

**Conclusion:**

Our results demonstrate that clinically relevant PAs are more common than usually suspected, especially prolactinomas and growth-hormone secreting PAs. These data highlight the need to increase the awareness of PAs, thereby enabling early diagnosis and treatment.

## INTRODUCTION

The prevalence estimates of pituitary adenomas (PAs) are inconsistent. According to epidemiological data derived from cancer registries, the prevalence of PAs is 25 cases per 100,000 inhabitants (1), and according to the most recent report from the Central Brain Tumor Registry of the United States, they account for approximately 15% of all brain tumors (2). On the other hand, postmortem studies have reported a mean prevalence of 11%, with the majority of tumors being microadenomas (3,4). With the widespread use of MRI, PA detection seems to have increased. In fact, in the meta-analysis by Ezzat and cols. (5), PAs were found in up to 22.5% of imaging studies. However, the findings of autopsy and imaging studies are not related to clinically relevant PAs but rather to asymptomatic tumors**,** yet clinically relevant PAs are associated with increased morbidity and mortality (6).

Recent epidemiological studies have shown that both the incidence (7-9) and prevalence (7,10-12) of PAs may have been previously underestimated.

The main purpose of this study was to estimate the incidence and prevalence rates of clinically relevant PA within the Hospital Italiano Medical Care Program (HIMCP), a well-defined population living in the urban and suburban area of the city of Buenos Aires. We defined clinically relevant PAs as those associated with endocrine dysfunction and/or mass effect.

## SUBJECTS AND METHODS

### Study setting

The study population was the members of a prepaid health maintenance organization, HIMCP, managed by a general, tertiary-level university hospital in Argentina (HIBA) that serves a community of over 150,000 members. Health care services are provided by physicians in two main hospitals and 24 peripheral outpatient medical clinics, located mainly in Buenos Aires’s inner city.

According to the 2010 Census, a total of 2,890,151 inhabitants live in Buenos Aires’s inner city, covering an area of 202 km^2^. Approximately 92% of this population is of white South European descent, and there is a minority of mixed native and other ethnicities (2010 Census. INDEC. Dirección General de Estadísticas y Censos. Argentina, http://www.indec.gov.ar) (13). Approximately 5% of this population is affiliated with the HIMCP.

Argentina’s health care system is maintained by three major providers: the state, the private sector and social security (the last two covering almost 18.3 million people, distributed among about 300 entities of varying scope and size). Beneficiaries of the private sector can freely choose their health maintenance organization.

The HIBA provides health services to two kinds of patients: patients affiliated with the HIMCP and patients belonging to other health providers sent to our hospital, as a tertiary center for evaluation. Only patients belonging to the HIMCP were included in the prevalence and incidence estimates. The patients are clearly identified by health provider, name, photograph, identification number and date of birth in the electronic database, thus preventing record duplication or misallocation. Before being admitted into the HIMCP, new patients must sign a sworn affidavit stating their pre-existing diseases and health conditions. A general practitioner will then perform a complete medical history and physical exam during the admission process.

A retrospective open cohort study was conducted that included all members of the HIMCP over 18 years old, with active memberships during the whole study period, from January 1, 2003, to January 1, 2014.

All medical care interventions including diagnostic studies – diagnostic laboratory tests and MRI imaging – were performed at HIBA and registered in a centralized electronic database.

### Data gathering

Cases of PA were identified by an exhaustive search in the HIMCP’s electronic database using the following Systematized Nomenclature of Medicine Clinical Terms (SNOMED-CT): acromegaly (Acro), Cushing’s disease (CD), prolactinoma, non-functioning adenoma (NFA) and thyrotropinoma. Related search terms (hyperprolactinemia, pituitary tumor, sellar or intrasellar tumor, pituitary adenoma) were also used. Every case among patients that was diagnosed and followed by endocrinologists, general practitioners, gynecologists, urologists, neurosurgeons and neuro-ophthalmologists was confirmed and classified by three trained staff endocrinologists. The PA subtypes were prolactinomas, NFAs, Acro, CD and thyrotropinoma. To ensure that no preexistent PAs were included as new cases, only those cases of patients with more than twelve months as members of the HIMCP were included to estimate the incidence rate.

A diagnosis of prolactinoma was established when serum prolactin levels were higher than 60 ng/mL in the presence of a pituitary tumor, and the patients showed therapeutic response to dopamine agonists. Patients with hyperprolactinemia without the presence of a pituitary tumor were not included. Acro was defined by levels of insulin-like growth factor type 1 (IGF1) above the reference range for age and gender, and unsuppressed GH in the oral glucose tolerance test, in the presence of a pituitary tumor. The diagnosis of CD was based on biochemical evidence of ACTH-dependent hypercortisolemia with unsuppressed ACTH levels (greater than 20 pg/mL) in the presence of a pituitary adenoma, or bilateral inferior petrosal sinus sampling showing a central: peripheral ratio > 2.0 or a post-desmopressin stimulation ratio > 3.0. NFA was diagnosed by the presence of a pituitary tumor not associated with clinical or biochemical evidence of hormone hypersecretion. Thyrotrophinoma was diagnosed if an inadequately normal or high TSH secretion was demonstrated in the presence of high free thyroxin levels and a pituitary adenoma. In the cases where surgery was performed, definitive diagnosis was based on pathological and immunohistochemical results. Patients were excluded from the analysis when the available data did not support a definitive diagnosis of PA after surgery or the specific diagnosis of non-adenomatous lesions was established by histopathological study.

Hormone-deficiency syndromes were defined following established criteria: the hypothalamic-pituitary-adrenal axis was considered impaired when the morning serum cortisol level (8-9 hours) was lower than 3 mcg/dL or the response to cosyntropin stimulation was lower than 18 mcg/dL. The pituitary-thyroid axis was deemed deﬁcient when the serum free and/or total thyroxin level was low for the reference range in the presence of normal or low TSH. In patients with normal serum prolactin levels, the hypothalamic-pituitary-gonadal axis was considered affected in men when their baseline circulating testosterone levels were below the reference range, in association with low or normal levels of follicle-stimulating (FSH) and luteinizing (LH) hormones. In women, this diagnosis was established when FSH levels were inadequately low in menopausal women, or when hypogonadotropic amenorrhea was detected in premenopausal women. The growth hormone axis was not explored in any of the patients in the series by means of an insulin hypoglycemia test or any other stimulating test.

Patient characteristics including age, gender and clinical presentation and diagnosis were recorded. Clinical features at presentation were classified as: 1) hormone excess, in cases with symptoms related to confirmed pituitary hormone excess; 2) mass effects, in cases with headaches and/or visual impairment; and 3) hypopituitarism, in cases with symptoms of confirmed pituitary-hormone deficiency. PAs without at least one of these three clinical features were considered clinically irrelevant and excluded.

All of the patients with incidentally found PAs were reviewed. If the patient’s medical record revealed any of the clinically relevant features mentioned above, even if previously undetected, the patient was also included as a case.

Regarding size, PAs with a maximal diameter of or greater than 10 mm were considered macroadenomas, while smaller ones were considered microadenomas.

### Statistical analysis

The incidence rates (IRs) were age standardized to the World Health Organization’s (WHO’s) 2000 standard population (SIR) (14) using a direct method. For SIR estimation, those patients with less than one year as members of the HIMPC were excluded to avoid the inclusion of prevalent cases. Unadjusted age-specific IRs were also estimated. All of the reported incidence rates are expressed per 100,000 members/year. We used rate ratios with their 95% confidence interval (95% CI) to compare sex-specific SIRs.

Prevalence was estimated by the Wilson method on January 1, 2014. For this purpose, members with PA fulfilling the inclusion criteria who were alive and belonged to the HIMCP at the time were included. All of the prevalence rates and 95% CIs are per 100,000 members.

The continuous variables are expressed as means and standard deviations (SD). The categorical variables are expressed as percentages of the total cases or groups.

### Ethics

This study complies with the tenets of the Helsinki Declaration and was approved by the Ethics Committee of the HIBA.

## RESULTS

**Figure 1:**
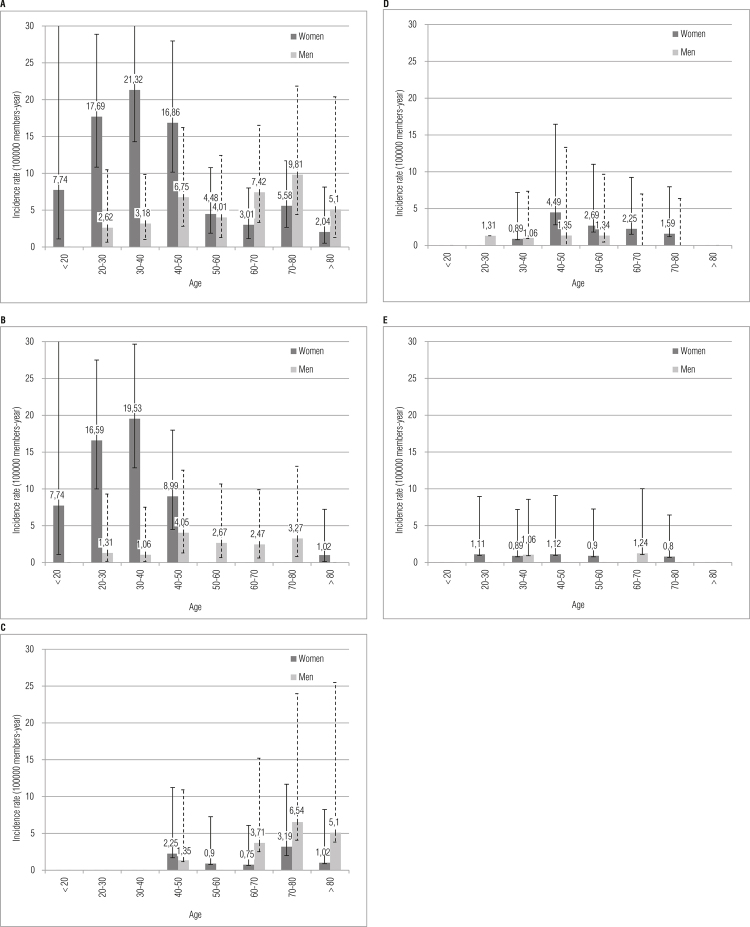
Overall and subtype pituitary adenomas incidence rate.

For the incidence estimates, 101 patients with PA matched our inclusion criteria within a population of 1,286,781.47 member-years at risk. The mean age at diagnosis of PA was 46.39 (18.2) yrs., with a predominance of female patients (n = 74; 73.3%) and the female patients being significantly younger (34.1 [0.9] vs. 53.9 [21.9]; p < 0.04). Sixty-one percent of the PAs were microadenomas (n = 62; 61.4%). The most common subtype was prolactinoma (57.43%) followed by NFA (18.81%), Acro (16.83%) and CD (6.93%). No thyrotrophinomas were diagnosed. The overall SIR was 7.39 (4.47-10.31). The female subjects had a significantly higher incidence than their male counterparts (5.85 vs. 1.54, respectively) with a SIR rate ratio of 3.79 (2.44-5.90) (Table 1). The incidence of PA increased with age in males, whereas the peak incidence among females was in the 30-40 age group, as shown in Figure 1A. Regarding clinical features at diagnosis, prolactinoma, Acro and CD mostly presented with signs and symptoms of hormone excess, while most patients with NFAs experienced mass effects and hypopituitarism. Further details regarding the incidence rates and clinical data of all of the PAs and subtypes are shown in Table 1.

**Table 1:** Incidence rates and clinical features in the 101 patients included for incidence estimates

	Total PA	Prolactinomas	NFA	ACRO	CD
Number of patients (%)	101 (100)	58 (57.43)	19 (18.81)	17 (16.83)	7 (6.93)
Mean age at diagnosis (SD)	46.39 (18.2)	37.5 (13.5)	68.7 (13.5)	51.5 (14.1)	47.2 (16.7)
Female patients (n) (%)	74 (73.3)	47 (81)	9 (47.4)	13 (76.5)	5 (71.4)
Microadenomas (n) (%)	62 (61.4)	49 (84.5)	1 (5.3)	6 (40)	6 (85.7)
Clinical features at presentation					
Hormone excess (n) (%)	81 (81)	58 (100)	-	16 (94.1)	7 (100)
Mass effects (n) (%)	23 (23)	4 (6.9)	16 (88.9)	2 (11.8)	1 (14.3)
Hypopituitarism n (%)	9 (9)	1 (1.7)	8 (44.4)	-	-
SIR (95%CI)	7.39 (4.47 – 10.31)	5.41 (2.57 – 8.25)	0.65 (0.31 – 0.99)	0.92 (0.44 – 1.41)	0.4 (0.08 – 0.73)
IR male (95%CI)	1.54 (0.9 – 2.18)	0.72 (0.26 – 1.17)	0.37 (0.12 – 0.62)	0.34 (0 – 0.67)	0,12 (0.05 – 0.29)
IR female (95%CI)	5.85 (3 – 8.69)	4.69 (1.89 – 7.5)	0.28 (0.05 – 0.51)	0.59 (0.23 – 0.94)	0.28 (0.01 – 0.56)
IR rate ratio (female/male) (95%CI)	3.79 (2.44 – 5.9)	6.56 (3.4 – 12.65)	0.75 (0.31 – 1.85)	1.75 (0.57 – 5.37)	2.39 (0.46 – 12.3)

IR and SIRs are expressed per 100,000 members/year. IR: incidence rate; SIR: standardized incidence rate; PA: pituitary adenomas; NFA: non functioning adenomas; Acro: acromegaly; CD: Cushing’s disease; SD: standard deviation; %: percentage; CI: confidence interval.

Incidence rate (IR) per 100,000 members/year according to 10 year age groups in male and females: < 20 years; ≥ 20 to < 30 years; ≥ 30 to < 40 years; ≥ 40 to < 50 years; ≥ 50 to < 60 years; ≥ 60 to < 70 years, ≥ 70 to < 80 years; ≥ 80 years.

**A:** All pituitary adenomas; **B:** Prolactinomas; **C:** Non-functioning adenomas; **D:** Acromegaly; **E:** Cushing disease.

**Figure 2:**
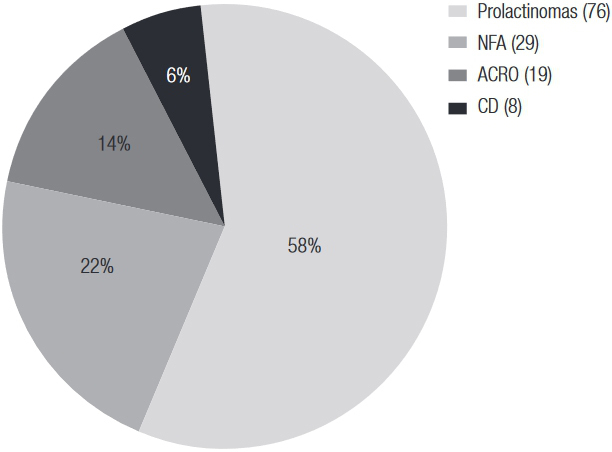
Distribution of pituitary adenoma subtypes for prevalence estimates in percentage (%) and number (n). NFA: non-functioning adenomas; Acro: acromegaly; CD: Cushing’s disease.

For the prevalence estimates, a total of 132 patients with PA were identified within the total population of HIMCP members alive on January 1, 2014 (135,019 adult members; 81,422 women and 53,597 men). The mean age at diagnosis of PA was 44.4 (17.2) yr, with a predominance of female patients (n = 102; 77.3%). Fifty-two percent of the PAs were microadenomas (n = 69, 52.3%). The most common subtype was prolactinoma (57.58%), followed by NFA (21.97%), Acro (14.39%) and CD (6.06%). No thyrotrophinomas were detected. The estimated prevalence rate was 97.76/100,000. A higher prevalence was found in the female patients: 125.27 (103.22-152.04) vs. 55.97 (39.21-79.89) in the male patients. Further details regarding prevalence estimates are shown in Table 2 and Figure 2.

**Table 2:** Prevalence rate and clinical features of the 132 patients included for prevalence estimates

	Total PA	Prolactinomas	NFA	ACRO	CD
Number of patients n (%)	132 (100)	76 (57.5)	29 (21.9)	19 (14.5)	8 (6.1)
Mean age at diagnosis (SD)	44.4 (17.2)	37.4 (14.2)	60 (16.7)	47.8 (13.2)	46.6 (15.3)
Female patients n (%)	102 (77.3)	63 (82.9)	19 (65.5)	14 (73.7)	6 (75)
Microadenomas n (%)	69 (52.3)	54 (70)	1 (3.4)	8 (42.1)	6 (75)
Clinical features at presentation					
Mass effects n (%)	34 (26.6)	8 (10.7)	24 (92.3)	2 (10.5)	0
Hormone excess	99 (77.3)	74 (98.7)	0	19 (100)	6 (75)
Hypopituitarism	7 (5.5)	1 (1.3)	6 (23.1)	0	0
Prevalence (95%CI)	97.76 (82.45 – 115.91)	56.29 (44.98 – 70.44)	21.48 (14.96 – 30.85)	14.07 (9.01 – 21.98)	5.93 (3 – 11.69)
Female prevalence (95%CI)	125.27 (103.22 – 152.04)	77.37 (60.49 – 98.97)	23.34 (14.94 – 36.45)	17.19 (10.24 – 28.86)	7.37 (3.38 – 16.08)
Male prevalence (95%IC)	55.97 (39.21 – 79.89)	24.26 (14.18 – 41.5)	18.66 (10.14 – 34.34)	9.33 (3.98 – 21.84)	3.73 (1.02 – 13.61)

All prevalences rates/100,000 members alive at 1^st^ January 2014. PA: pituitary adenomas; NFA: non-functioning adenomas; Acro: acromegaly; CD: Cushing’s disease; SD: standard deviation; %: percentage; CI: confidence interval.

### Prolactinomas

Prolactinomas had the highest SIR: 5.41 (2.57-8.25). The mean age at diagnosis was 37.5 (13.5) yr, with a very high proportion of females (81%), most of them having microadenomas (84.5%). Microadenomas were more frequent in female vs. male patients: 53 (72.6%) and 9 (34.6%), respectively (p < 0.001). Patients with microadenomas were also younger than those with macroadenomas: 38 (12.8) vs. 60.1 (17.3) yr (p < 0.05). The highest IR for females was reached in the 3-40 age group, whereas no significant incidence peak was found among the male patients (Figure 1B).

Prolactinomas had also the highest prevalence rate: 56.29 (44.98-70.44)/100,000; 83% were harbored by women, and 70% were microadenomas (Table 2).

#### Non-functioning adenomas

The SIR of NFAs was 0.65 (0.31-0.99). The mean age at diagnosis was 68.7(13.5) yr, which was significantly higher than that for prolactinomas, and 52.6% occurred in males. All of the NFAs but one were macroadenomas (94.7%).

Although the SIR was higher in males, this difference was not statistically significant (Table 1). NFAs showed the peculiar feature of increasing their incidence with age, especially in males, with the highest IRs attained in the 70-80 age group for both genders (Figure 1C).

The prevalence rate for NFAs was 21.48 (14.96-30.85)/100,000; 65.4% were harbored by female patients, and all but one were macroadenomas (Table 2).

#### Acromegaly

The SIR for Acro was 0.92 (0.44-1.41), the mean age at diagnosis was 51.5 (SD 14.1) yr and 76% of the patients were female. Sixty percent were macroadenomas. The highest IR in women was reached in the 40–50 age group, with no distinctive peak of incidence in males (Figure 1D). Although the SIR was higher in females, this difference was not statistically significant (Table 1).

The prevalence rate was 14.07 (9.01-21.98)/100,000. They occurred mostly in women (73.7%) and were macroadenomas in 57.9% of the cases (Table 2).

#### Cushing’s disease

The SIR for CD was 0.4 (0.08-0.73). The mean age at diagnosis was 47.2 (SD 16.7) yr, and most of them were microadenomas (85.7%). Although females had higher SIRs, this difference was not statistically significant (Table 1).

The prevalence rate was 5.93 (3-11.69)/100,000; 75% were harbored by females, and 76.6% were microadenomas (Table 2).

## DISCUSSION

This is the first study to estimate the prevalence and incidence of pituitary adenomas in Latin America. In this retrospective study, we found high incidence and prevalence of clinically relevant PA. Most of them were prolactinomas, predominantly microadenomas in female and younger patients. The incidence of PAs found in our study is higher than those reported by other authors, and the overall prevalence is similar to that published in Belgium, which in turn was the highest population-based prevalence ever published. NFAs were less frequent than in other reports, whereas acromegaly showed a prevalence rate in keeping with other published series but a higher incidence.

Some epidemiological studies of PA have been published in the last few years. In three of them (7-9), the SIR was estimated at about 4/100,000/year, which is significantly higher than previously reported (6). In three other studies (10-12), the reported prevalence of PAs was significantly higher than the one previously estimated from cancer registries (1), in agreement with our results.

To the best of our knowledge, only one study (7) estimates both the incidence and prevalence rates of PAs, like ours. Both epidemiological outcome measures are important for assessing the real burden of these tumors on health care resources because although they are benign, they are an important cause of morbidity and mortality.

The first aspect to discuss regarding our results is the high SIR [7.4 (4.47-10.31)/100.000 members/year], which appears to be higher than previously published results. However, the 95% CI overlaps those of Malta [4.27 (3.7–4.9)] (7) and Finland [3.98 (3.37-4.6)] (8), and are only significantly higher than those reported in Sweden [3.9 (3.6-4.3)] (9).

The prevalence rate of PA in our cohort was 97/100,000, or 1 PA for every 1,030 individuals, which is very similar to that reported by Liege (94/100.000, 1 PA/1,064 individuals) (10), and slightly higher than other reports that estimate the prevalence of PAs between 75 and 80/100,000 (7,11,12).

The overall SIR, as clearly depicted in Figure 2, mainly arises as a result of the high incidence of prolactinomas [5.41 (2.57-8.25)], which accounted for 57% of all of our PAs. Although not significantly higher than that of Finland [2.16 (1.70-2.63)] (8), our incidence of prolactinomas is higher than those of Malta (7) and Sweden (9). This could be attributed to the fact that our study included patients diagnosed and treated not only by endocrinologists and neurosurgeons but also by gynecologists and general practitioners, who usually care for patients with microprolactinomas in our health program. As put forward by the authors of the Swedish series, different inclusion criteria can alter SIR estimates: the relatively low incidence of prolactinomas and high incidence of NFAs in their study has been attributed to the inclusion of PAs from a register that mainly captures cases reviewed by endocrinologists. Our case-finding strategy of using all of the subspecialties that diagnose and care for PAs as a source of incidence data could be revealing a more complete picture, and rendering a more valid estimate of incidence, than endocrinology department-based studies, especially for prolactinomas*.* Moreover, the prevalence of prolactinomas in our study, 56.3/100,000, is also very similar to that of the Belgian study (66/100,000), which also included cases treated by general practitioners and those from other medical subspecialties besides endocrinologists (10).

Of all of the tumor subtypes, significant female gender predominance was found only in prolactinomas that were clearly more prevalent in women (81%) in our cohort. Prolactinomas also affected younger females, and most were microadenomas, showing the gender differences described by several authors (15-17).

NFAs were the second-most-frequent tumor subtype, at 18% of our incidence cohort; they were associated with either hypopituitarism or mass effects and, in contrast to prolactinomas, were mainly macroadenomas and did not show a gender predominance. NFAs were less frequent in our study, although the SIR [0.65 (0.31-0.99)] was similar to the one reported in Finland [1.02 (0.86-1.19)] (8), and the age of peak incidence was between the fifth and seventh decades. This can be attributed to the fact that when we excluded clinically irrelevant tumors, many were NFA microadenomas. The findings are further supported by the high proportion of macroadenomas, 95%, when compared to other series that quote 64% and 82%, respectively (9-11). Our prevalence of NFAs was 21.48/100.000, which is in agreement with the prevalence reported by other epidemiological studies between 14/100,000 (10) and 26/100,000 (7).

Interestingly, the incidence of Acro in our cohort appears to be high [0.92 (0.44-1.41)] and is only comparable to the one reported in Malta [0.31 (0.19-0.53)] (7). In the same way, the prevalence of Acro in our study (14.07/100,000) was also considerably higher than previously estimated (4 /100,000) (18), but similar to those of Malta (12.5/100,000) (7) and Liege (12.2/100.000) (10). However, it was lower than the one estimated when screening for elevated IGF1 levels in a primary care setting, in a cross-sectional study from Germany (19), and almost half the one reported in a recent study from Belo Horizonte, based on screening with a questionnaire that assessed the enlargement of extremities (20). All of these reports suggest that the prevalence of growth-hormone-secreting pituitary adenomas has been heretofore underestimated. This is important from the perspective of healthcare resource allocation, as most patients with acromegaly harbor macroadenomas and surgery may not result in remission of the disease. Prolonged treatment with somatostatin analogs and/or pegvisomant is costly, and an adequate estimation of the prevalence and incidence of Acros will allow for an accurate assessment of its burden on healthcare resources.

The SIR of CD found in our study, 0.40 (0.08-0.73), is higher but not significantly different from that found in previous studies: 0.07 (0.03-0.21) to 0.18 (0.11-0.25) (7-9). Nonetheless, it is lower than the incidence found in commercially insured patients under 65 years old in the United States (8 cases per million/year) that was recently published (21). CD is difficult to diagnose, so larger cohorts of patients are likely needed to estimate the true prevalence and incidence of this disease.

The HIBA is a tertiary care center. However, it is important to underscore that our results were not influenced by referral bias, since only patients belonging to the HIBA health program, HIMCP, were included, per our stringent inclusion criteria. Observer and selection bias were also precluded by including only members who did not have a PA on admission and did not develop it during the twelve months following their affiliation, further strengthening our results. Furthermore, the similarities of our results to those reported by other investigators, especially those for PA in the well-defined area of Liege, suggest that no significant selection bias occurred in our study.

The main weakness of our study is that it refers to a specific Buenos Aires population belonging to a prepaid medicine program, and not a geographically defined population like Malta or Liege. Nevertheless, the population cared for by our hospital network is numerically larger than those of many other published series; it includes approximately 5% of the population of Buenos Aires and is thus a representative sample. Although patients from outside Buenos Aires and its suburbs may also be included in the health care program, over 150,000 members have valid addresses within the city’s limits.

The main strengths of our study are the inclusion of different specialists like gynecologists and general practitioners in the central medical record database, the use of diagnostic criteria in agreement with international standards, access to individual patient data and the confirmation of the diagnoses by one of the three neuroendocrinology specialists in our group. The centralized medical records in our medical center include all lab and imaging studies performed in-house, minimizing data loss, and allocation errors.

In conclusion, our results demonstrate that clinically relevant pituitary adenomas are more common than usually suspected, especially prolactinomas and growth-hormone secreting PAs. These data highlight the need to increase awareness of PA, thereby enabling early diagnosis and treatment of these tumors, which mainly affect the economically active population and are associated with increased morbidity and mortality.
